# Safe sleeping positions: practice and policy for babies with cleft palate

**DOI:** 10.1007/s00431-017-2893-0

**Published:** 2017-03-22

**Authors:** Karen Davies, Iain A Bruce, Patricia Bannister, Peter Callery

**Affiliations:** 1 Division of Nursing, Midwifery and Social Work, University of Manchester, Jean Mc Farlane Building, Oxford Road, Manchester, M13 9PL UK; 2 Paediatric ENT Department, Royal Manchester Children’s Hospital, Central Manchester University Hospitals NHS Foundation Trust, Manchester Academic Health Science Centre, Manchester, M13 9WL UK; 3 Respiratory and Allergy Centre, Institute of Inflammation and Repair, Faculty of Biology Medicine and Health, University of Manchester, Manchester, UK; 4 Cleft and Craniofacial Clinical Research Centre, Division of Dentistry, University of Manchester, Manchester, M15 6FH UK

**Keywords:** Safe sleeping positions, Infants, Cleft palate

## Abstract

Guidance recommends ‘back to sleep’ positioning for infants from birth in order to reduce the risk of sudden infant death. Exceptions have been made for babies with severe respiratory difficulties where lateral positioning may be recommended, although uncertainty exists for other conditions affecting the upper airway structures, such as cleft palate. This paper presents research of (i) current advice on sleep positioning provided to parents of infants with cleft palate in the UK; and (ii) decision making by clinical nurse specialists when advising parents of infants with cleft palate. A qualitative descriptive study used data from a national survey with clinical nurse specialists from 12 regional cleft centres in the UK to investigate current practice. Data were collected using semi-structured telephone interviews and analysed using content analysis. Over half the regional centres used lateral sleep positioning based on clinical judgement of the infants’ respiratory effort and upper airway obstruction. Assessment relied upon clinical judgement augmented by a range of clinical indicators, such as measures of oxygen saturation, heart rate and respiration.

*Conclusion*: Specialist practitioners face a clinical dilemma between adhering to standard ‘back to sleep’ guidance and responding to clinical assessment of respiratory effort for infants with cleft palate. In the absence of clear evidence, specialist centres rely on clinical judgement regarding respiratory problems to identify what they believe is the most appropriate sleeping position for infants with cleft palate. Further research is needed to determine the best sleep position for an infant with cleft palate.

**Table:** 

**What** **is Known** • *Supine sleep positioning reduces the risk of sudden infant death in new born infants.* • *There is uncertainty about the benefits or risks of lateral sleep positioning for infants with upper airway restrictions arising from cleft palate.* **What is New** • *Variability exists in the information/advice provided to parents of infants with cleft palate regarding sleep positioning.* • *Over half the national specialist centres for cleft palate in the UK advise positioning infants with CP in the lateral position as a routine measure to reduce difficulties with respiration.*

## Introduction

A number of risk factors contribute to sudden and unexpected death in infants [16, 33]. A well-evidenced risk factor is the infant’s sleeping position during the first few months of life. Guidance in the UK, and internationally, recommends that parents should place their babies in the supine position for sleep [30]. In general, ‘back to sleep’ campaigns have been associated with continuing reductions in unexpected infant deaths [10]. There is evidence of significantly increased risks associated with the alternative positions of prone and lateral positioning [2, 26, 31]. However, the guidance gives little indication that certain conditions may benefit from an alternative sleeping position and evidence about exceptions to the standard advice is limited. Advice for families of infants with a cleft palate (CP) is therefore variable. Previous research suggests that a supine position may not be appropriate for infants with Pierre Robin sequence (PRS). In this condition, lateral or prone positioning may be recommended [11] with some evidence suggesting that prone positioning can relieve respiratory difficulties in 70% of cases [34]. Other researchers acknowledge that the prone position may mask severe breathing difficulties and recommend close monitoring [25]. In the absence of definitive evidence, advice on sleeping positions can be confusing for parents of children with CP and health professionals, as illustrated by the following comment from a parent recorded on a UK online forum [5] (posted 23/01/2011):

I forget why they say to put them on there [sic] side; I think it’s to do with there [sic] tongue and the cleft.

Isolated cleft palate is a congenital condition affecting the structure of the hard and/or soft palate [6], and is commonly associated with differences in tongue position, upper airway structure and functioning [24]. All children affected by this condition tend to have smaller upper airways [21] and are at greater risk of sleep-disordered breathing (SDB) and obstructive sleep apnoea (OSA) [6, 14, 22, 23]. Periods of partial or complete upper airway obstruction during sleep are often hard to detect but can be associated with both immediate and chronic changes in blood pressure, heart rate and cardiac function [15]. This is thought to be underreported in children with CP [27], despite the possibility that they may be up to five times more likely to be at risk of OSA than unaffected children [23]. Moreover, associations between SDB, poorer cognitive outcomes and ‘failure to thrive’ suggest there is potential for impact on longer term outcomes [3, 18].

Few studies have evaluated SDB in children less than a year [17]. However, sleep positioning is used as a simple therapeutic intervention to relieve SDB in cases of obvious airway compromise, such as Pierre Robin sequence (PRS) [8]. In these examples, upper airway obstruction is more evident in the supine position and is believed to improve in lateral and prone sleep positions. However, research studies are limited, with evidence drawn from expert opinion and single-centre evaluations [4]. The advantages or risks of different sleep positions have yet to be demonstrated using polysomnography recordings with infants with CP [8]. This raises an important question of whether babies with an isolated CP should be subject to the same advice about sleep positioning as unaffected babies or could they benefit from a lateral sleep position? The controversy exists because of the extensive research supporting supine positioning in unaffected children, and dearth of evidence exploring supine or lateral sleep positioning in the CP population. Consequently, specialist practitioners in the UK have identified variation in practice between the 12 centralised cleft centres that offer specialist care for all infants with CP, creating uncertainty for parents and discrepancy in clinical practice. To our knowledge, there is no published information about usual practice with infants with CP in other countries. In order to investigate the use of sleep positioning as a therapeutic intervention with a CP population, we undertook a national survey of practice in the UK.

This paper presents the findings of a survey of current practice in advising parents and health professionals on sleeping positioning for infants with CP under the age of 6 months in the UK. It reports a qualitative survey conducted as part of a wider research programme on sleep positioning conducted [12].

## Methods

A qualitative survey was used to gather information about current practice in advising sleep positioning for infants with both complete and incomplete CP. Qualitative surveys offer a systematic method to collect descriptive evidence of practice [19]. Qualitative description is used in health research to provide a rich description of events or practice, founded on existing knowledge and clinical experience of researchers [28]. The descriptive categories derived from analysis are often formulated using participants’ own language rather than more abstract themes based on interpretive meaning assigned by researchers. A qualitative researcher collected data from clinical nurse specialists (CNS) working in specialist cleft centres throughout the UK. The paper adopts the COREQ standards for reporting [35]. The descriptive study formed part of a larger feasibility study, investigating sleep positioning, approved through NHS Ethics (NRES Committee North West—Greater Manchester Central 15/NW/0010). Separate ethical approval was not required for the descriptive study of current practice as it was judged to be a service evaluation by the ethics board [13].

### Aims

The aims of the study were (i) to describe current advice on sleep positioning provided to parents of new born infants with CP by cleft centres in the UK; and (ii) to explore how decisions about sleep positioning are made by specialist nurses.

### Participants

CNS from 13 national centres in the UK were invited to participate in the qualitative survey. Sampling was purposive, aiming to survey practice in all 13 regional cleft centres in the UK through semi-structured interviews with CNS who are ideally placed to provide information about the advice provided for sleep positioning in cleft centres. They assess new born infants and advise on the management of cleft palate soon after diagnosis. They provide information, support and on-going management advice to parents and health professionals, identifying any deterioration in health and making appropriate referrals for medical input where necessary. They are experienced clinicians working in paediatric care with additional professional skills in managing infants with cleft palate, acquired through professional development as part of the Cleft and Craniofacial Clinical Excellence Network in the UK [9].

### Data collection

Data were collected during April 2015 by an experienced qualitative researcher with a clinical background (KD). An expert in cleft nursing (PB) discussed the research with participants from the Cleft and Craniofacial Clinical Excellence Network and gained agreement for the researcher to contact them. The researcher contacted each CNS by telephone, explained the purpose of the study and invited them to complete a semi-structured telephone interview. Informed verbal consent was secured from each participant. Each interview took place while the CNS was in their work place. Each participant was asked the same closed questions about current practice and open questions to qualify, explain and elaborate on their responses, using a topic guide prepared with the guidance of an advisory group of researchers and cleft specialists (Table 1). Participants were encouraged to give full responses, and the duration of each interview was approximately 30–40 min. The interview was divided into three sections: (i) giving parents of babies with cleft palate advice about sleep positions, (ii) identification and management of airway obstruction and (iii) professional views. The questions referred to all infants diagnosed with cleft palate, but participants were encouraged to focus their responses on general advice rather than the airway management of syndromes such as PRS. The interviews were audio recorded and descriptive notes were written during and after each interview.

**Table 1:** Topic guide for semi-structured telephone interviews

Topic guide for semi-structured interviews with clinical nurse specialists	
Section 1: giving parents of babies with cleft palate advice about sleeping position	
In your experience, do parents have a clear idea about what is the best sleeping position for their baby with CP? What advice is provided by you in your centre about sleeping position to parents of an infant with a cleft palate? Why? How is the information given to parents and clinicians? (written, verbal, webpage?) Are there any exceptions in providing this advice? Which patients would the exceptions apply to? Are parents told that it is not clear what the best sleeping position for infants with cleft palate is? Are you aware of any contradictions between the advice that parents receive? Any conflict between the advice and parents’ own beliefs? Are you aware of any difficulties parents experience in following advice? What influences the decision you make about recommending a sleeping position? How do you balance the contradictory advice parents are given?	
Section 2: identification and management of airway obstruction	
How significant a problem is airway obstruction in infants with cleft palate? How do you assess babies breathing difficulties and grade their difficulties before giving advice? When are children first screened for airway obstruction and how often is this repeated there after? What approaches are used in your centre to screen for airway obstruction? In your experience, how do parents respond to airway problems in an infant with a cleft palate? How concerned are (i) parents and (ii) other clinicians such as paediatricians, GPS, and health visitors?	
Section 3: professional views	
How concerned are you about the advice given to parents about sleeping position? Would you be interested in supporting a study to investigate this further? Would you have any specific concerns about this and what would reassure you?	
Section 4: additional comments	

### Data analysis

Data were analysed using content analysis by two researchers (KD and PC), in keeping with qualitative methods [28]. Closed question responses were tabulated and open responses were coded into descriptive categories derived inductively as the analysis progressed. A framework was devised using the descriptive categories to map the information collected from each participant and provide an overview of practice from the centres. Interpretation of the findings was reviewed with an advisory group including researchers, cleft and respiratory clinicians and service users.

## Results

Surveys were completed with CNS at 12 specialist centres for CP with only one centre declining to be interviewed. Each CNS reported the policy and practice of their entire centre, and therefore the results provide comprehensive evidence of practice across most of the UK.

### Over half the specialist regional centres routinely use lateral positioning

The advice for parents on sleeping position varied between the cleft centres: seven used lateral positioning and five used supine positioning as their usual practice for infants with cleft palate. However, in those centres using supine positioning, advice was conditional on how well infants tolerated this position. Recommendations fell into two groups:

i. Supine positioning as a matter of course, assuming that babies with cleft palate should be treated identically to non-affected babies (five centres).
ii. Lateral positioning as a matter of course, assuming that all babies with cleft palate will experience SDB that can be managed through lateral positioning (seven centres).

Advice was described by some as ‘a matter of course’, but CNS in the group using supine positioning explained how this could then be modified following observation of a baby’s breathing and comfort in sleeping. They reported advising a lateral sleeping position for babies with respiratory distress, even when the underlying approach in the centre was to recommend supine positioning.

The participants explained the rationale for their centre’s advice. Those who used a supine position sought to follow national guidance for all infants. They emphasised the importance of normalising babies with cleft palate *unless* they showed signs of airway obstruction. Parents could subsequently be advised to change to a lateral sleeping position as a first stage management for visible symptoms of respiration difficulties. Centres using lateral positioning as a ‘matter of course’ from the outset regarded cleft palate as an immediate indicator for potential breathing difficulties, believing that intermittent clinical signs of upper airway obstruction were common especially after oral feeds. Lateral positioning was used as a precautionary management approach.

All CNS commented on the importance of using their specialist knowledge, experience and training to determine their practice. Although CNS expressed confidence in their clinical judgement in recommending the most appropriate sleep position, 9 out of 11 specifically expressed concern about the lack of clinical evidence relating to sleep positioning and identified a need for further research.

### Clinical observations of breathing difficulties determine advice

All centres use lateral positioning for infants demonstrating respiratory distress. CNS commented that the critical feature for decision making was the existence of breathing difficulties: if an infant showed evidence of upper airway obstruction, then they suggested a lateral sleeping position as the first line management. They observed whether this reduced the symptoms in infants when they were awake, during and after an oral feed and during sleep.

CNS reported that respiratory difficulties can be challenging to identify in new born infants, with changes emerging as infants are handled, orally fed or as environmental circumstances change. There was considerable consistency between CNS about the way infants were monitored. Oxygen saturation levels were the most frequently reported clinical indicator (nine centres), followed by noisy breathing (six centres) and higher respiratory/heart rates (five centres). Although several respondents commented on capillary carbon dioxide as an important indicator of upper airway obstruction, it was not reported to be monitored routinely.

Most centres used overnight and daytime pulse oximetry, which is a non-invasive procedure that measures oxygen saturation. Pulse oximetry may form part of the monitoring process in infancy for infants considered at risk of breathing disorders, and is undertaken in the community or during a hospital admission. However, this does not provide a comprehensive assessment of the amount of oxygen used by an individual and does not measure the level of carbon dioxide in the blood. Sleep studies using pulse oximetry predominantly took place at home (six centres) and were not necessarily undertaken by the cleft team. Three centres reported that other teams, such as the respiratory team and community paediatric nurses, were responsible for providing this service. Detailed evidence of airway obstruction based on measures of oxygen, carbon dioxide, respiratory effort, heart rate and airflow, using infant polysomnography, was available at five centres. Three CNS reported that their centre experienced specific difficulties undertaking any form of sleep studies, such as variation in access to sleep studies across a region, limited availability of pulse oximetry machines and unwillingness of paediatricians to initiate a referral for sleep studies.

### CNS support parents in adopting appropriate sleep positions

CNS in centres advising lateral positioning referred to explaining to parents the anatomical differences between babies with and without cleft palate. They discussed how this may affect breathing and advised on management. The intention of providing a rationale for parents was to (i) prioritise the baby’s safety through a sleeping position that was believed to decrease upper airway obstruction; (ii) reassure parents about using an alternative sleeping position which was a counter to general guidance; and (iii) give them confidence to explain the rationale to generic health professionals, such as health visitors, who may be reluctant to endorse advice inconsistent with official guidance.

CNS did not routinely provide written advice to parents about sleep positioning. Occasionally, they referred to written guidance as part of an individualised management plan.

### Care pathways and grading systems not consistently used

Protocols of care and care pathways are frequently used in multidisciplinary teams to ensure consistent service delivery, although reference to sleep positioning and airway management in the early weeks of life for CP are not usually specified. Six centres referred to locally agreed written protocols and pathways that could be used for informing decision making about airway management. The extent of their use, and relevance to sleep positioning, was not clear from the responses. Two CNS referred to an implicit use of pathways, mentioning that they followed a pathway as part of their clinical reasoning, but not necessarily using a written protocol common to the whole team.

Similarly, there was little consistency in the use of grading systems to categorise the severity of the respiratory difficulties. Fewer than half the centres (three centres) reported using an agreed grading system. Those that used them referred to locally designed and agreed systems.

## Discussion

This study identifies variation in policy and practice provided by CNS about sleeping positions for infants with cleft palate using a national survey of centres, highlighting the continuing uncertainty about the best practice regarding sleep positioning. Over half the centres in the study advise lateral positioning, based on the belief that this reduces respiratory effort and the risks associated with SDB. The other centres advise supine positioning in line with national guidelines to reduce the risk of sudden infant death. All centres make adjustments if obvious signs of increased respiratory effort are apparent. The variation indicates uncertainties that arise from the limited, and at times, confusing research evidence available to date. There is evidence that prone positioning can reduce respiratory obstruction in babies with PRS [20]. Very few studies have investigated SDB in infants with cleft palate only [32]. OSA has potential long-term effects on children’s health and development and prevention is important because treatment options are limited [7]. However, there is uncertainty about its prevalence [32] and observable symptoms of SDB do not distinguish between babies with and without OSA [7, 32].

CNS referred to the lack of definitive evidence to support their clinical decision making about sleep positioning, and so use a range of clinical indicators to inform the advice they provide. These were principally oxygen saturation, noisy breathing and higher respiration/heart rate. There was considerable variation in accessing more detailed assessments, such as polysomnography, which may also reflect the lack of consensus found in recent research studies about the most reliable way to measure OSA [17, 32].

The results of the study illustrate the clinical dilemma that practitioners face when recommending sleep positioning for infants with cleft palate. The uncertainty between recommending supine positioning, in order to reduce the risk of sudden infant death and lateral positioning to reduce breathing difficulties, remains unresolved. Parents of infants with cleft palate have significant practical and emotional challenges in learning to care for their infant [29]. The implications of variable advice regarding sleep positioning and the associated risks that may accompany either recommended position may add to parents’ anxiety. There is clearly a tension between adhering to national guidance on sleeping position [33] and applying professional judgement to the clinical signs of respiratory difficulties which cannot easily be resolved without detailed research based on monitoring infants in alternative sleeping positions.

Three limitations of the research need to be considered. First, the results describe current practice as reported by CNS. The open-ended approach allowed interviewees to raise issues as the study progressed but there were not always opportunities to discuss them with participants in earlier interviews. While access to sleep studies emerged as an important concern for some interviewees, we were unable to collect systematic data about the organisation of access to sleep studies in each centre. Some CNS raised issues about whether advice should be provided through home visits or telephone reviews with parents, but detailed data about service design, organisation and delivery were beyond the scope of this study.

Second, this descriptive survey of current practice nationally could not capture the full complexity and range of factors influencing the process of clinical reasoning and decision making by CNS in hospital and home settings.

Finally, some clinicians reported working with parents to enable them to feel confident in recognising breathing difficulties and making their own decision about positioning their baby. This study was not designed to explore how the process of parent education and support influenced parents’ choices about positioning their infants [1].

## Conclusion

Sleep positioning for vulnerable infants is a controversial and unresolved area of infant care [10]. There is growing evidence linking both prone and lateral sleep positioning with increased risks of sudden infant death [2, 26]. National guidance in the UK recommends adopting the supine position in order to reduce this risk. However, the benefits and risks of using lateral sleep positioning for infants with conditions known to be associated with upper airway obstruction are uncertain, and use with infants with cleft palate is anecdotal. This paper reviews the advice currently provided for parents of infants with cleft palate about safe sleeping positions by all but one of the regional specialist cleft centres in the UK. Over half the centres use lateral positioning, but the uncertainty of evidence to support this practice is a matter of concern for CNS in cleft centres. The variation in advice indicates a lack of research evidence to guide practice. Uncertainty about practice that differs from the standard advice of generalist health professionals including midwives, health visitors and general practitioners has the potential to cause anxiety for parents and risks for infants. Research is needed to provide an evidence base for practice to minimise the risks of OSA as well as sudden death in infants with cleft palates.

## Abbreviations

CP: Cleft palate
CNS: Clinical Nurse Specialist
OSA: Obstructive Sleep Apnoea
PRS: Pierre Robin sequence
SDB: Sleep-Disordered Breathing
UK: United Kingdom
